# PECSO: An Improved Chicken Swarm Optimization Algorithm with Performance-Enhanced Strategy and Its Application

**DOI:** 10.3390/biomimetics8040355

**Published:** 2023-08-10

**Authors:** Yufei Zhang, Limin Wang, Jianping Zhao

**Affiliations:** 1 School of Computer Science and Technology, Changchun University of Science and Technology, Changchun 130022, China; 2019200107@mails.cust.edu.cn; 2 School of Information Science, Guangdong University of Finance and Economics, Guangzhou 510320, China

**Keywords:** chicken swarm optimization, free grouping mechanism, niche technology, spiral learning strategy, synchronous update, optimization problems

## Abstract

To solve the problems of low convergence accuracy, slow speed, and common falls into local optima of the Chicken Swarm Optimization Algorithm (CSO), a performance enhancement strategy of the CSO algorithm (PECSO) is proposed with the aim of overcoming its deficiencies. Firstly, the hierarchy is established by the free grouping mechanism, which enhances the diversity of individuals in the hierarchy and expands the exploration range of the search space. Secondly, the number of niches is divided, with the hen as the center. By introducing synchronous updating and spiral learning strategies among the individuals in the niche, the balance between exploration and exploitation can be maintained more effectively. Finally, the performance of the PECSO algorithm is verified by the CEC2017 benchmark function. Experiments show that, compared with other algorithms, the proposed algorithm has the advantages of fast convergence, high precision and strong stability. Meanwhile, in order to investigate the potential of the PECSO algorithm in dealing with practical problems, three engineering optimization cases and the inverse kinematic solution of the robot are considered. The simulation results indicate that the PECSO algorithm can obtain a good solution to engineering optimization problems and has a better competitive effect on solving the inverse kinematics of robots.

## 1. Introduction

Swarm intelligence algorithms have been widely recognized since they were proposed in the 1990s [1]. They have a simple structure, good scalability, wide adaptability and strong robustness. Based on different biological habits and social behaviors, scholars have proposed numerous swarm intelligence algorithms, such as particle swarm optimization algorithm (PSO) [2], genetic algorithm (GA) [3], bat algorithm (BA) [4], social spider optimizer [5], CSO algorithm [6], moth–flame optimization algorithm (MFO) [7], whale optimization algorithm (WOA) [8], marine predators algorithm [9], battle royale optimization algorithm (BRO) [10], groundwater flow algorithm [11], egret swarm optimization algorithm [12], coati optimization algorithm [13], wild geese migration optimization algorithm [14], drawer algorithm [15], snake optimizer [16], fire hawk optimizer [17], etc. The algorithms are studied in terms of parameter set, convergence, topology and application. Among them, the CSO algorithm is a bionic swarm intelligent optimization technology proposed by Meng et al., named after the foraging behavior of chickens. Its main idea is to construct a random search method by simulating the behavior of roosters, hens and chicks in a chicken flock. On this basis, the optimization problem is solved through three chicken position update equations in the hierarchy. The principle of this algorithm is simple and easy to implement.

The algorithm has the advantages of simple principles, easy implementation and simple parameter setting. It has been widely used in the fields of trajectory optimization, economic dispatching, power system, image processing, wind speed prediction [18], and so on. For example, Mu et al. [19] used the CSO algorithm to optimize a robot trajectory for polishing a metal surface. The target of the optimization is to minimize the running time under kinematic constraints such as velocity and acceleration. Li et al. [20] applied a CSO algorithm to hypersonic vehicle trajectory optimization. Yu et al. [21] proposed a hybrid localization scheme for mine monitoring using a CSO algorithm and wheel graph, which minimized the inter-cluster complexity and improved the localization accuracy. Lin et al. [22] designed a CSO algorithm (GCSO) based on a high-efficiency graphics processing unit, which increased the population diversity and accelerated the convergence speed through parallel operations.

With the continuous expansion of application fields and the increase in problem complexity [23], the CSO algorithm has shown some deficiencies in solving these complex, high-dimensional problems, including low convergence accuracy and poor global exploration ability [24]. To solve these problems, many scholars have improved it, and now there are many variants of the CSO algorithm. In terms of initializing the population, the diversity of the population is enhanced by introducing a variety of strategies, such as chaos theory, mutation mechanisms, elimination–dispersion operations and deduplication factors [25,26], which are more conducive to finding the optimal solution to the problem. However, most means of improvement have been designed for individual update methods. Meng and Li [27] proposed to improve the CSO algorithm by using quantum theory to modify the update method of chicks. The algorithm was applied to the parameter optimization of the improved Dempster–Shafer structural probability fuzzy logic system, achieving good results for wind speed forecasting. Wang et al. [28] introduced an exploration–exploitation balance strategy in the CSO algorithm; 102 benchmark functions and two practical problems verified its excellent performance. Liang et al. [29] innovated an ICSO algorithm by using Lévy flight and nonlinear weight reduction to verify its outstanding performance in robot path planning. The other type is hybrid meta-heuristic algorithms, the combination of CSO with other algorithms. Wang et al. [30] provided an effective method to solve the multi-objective optimization problem based on an optimized CSO algorithm. The improved scheme includes dual external archives, a boundary learning strategy and fast, non-dominated sorting, and its superior performance has been verified by 14 benchmark functions. Li et al. [31] introduced the information-sharing strategy, spiral motion strategy and chaotic perturbation mechanisms into the CSO algorithm, which improved the identity of the photovoltaic model’s parameters. In addition, the combination of multiple algorithms is also a hot research topic. Deore et al. [32] integrated a chimp–CSO algorithm into the training of the network intrusion detection process. Torabi and Safi-Esfahani [33] combined the improved raven roosts optimization algorithm with the CSO algorithm to solve the task scheduling problem. Pushpa et al. [34] integrated a fractional artificial bee–CSO algorithm for virtual machine placement in the cloud. These hybrid algorithms have proved to have superior computational performance.

In summary, the CSO algorithm outperforms many naturally inspired algorithms for most benchmark functions and when solving practical problems. However, the “no free lunch theorem” shows that it is of great significance to further research the improvement of the CSO algorithm [35]. Therefore, an improved CSO algorithm with a performance-enhanced strategy is proposed, named the PECSO algorithm. It introduces the free grouping mechanism, synchronous updates and spiral learning strategy. The position updating method of the roosters, hens and chicks is redesigned.

The main contributions of this paper are summarized as follows:

1. A hierarchy using a free grouping mechanism is proposed, which not only bolsters the diversity of individuals within this hierarchy but also enhances the overall search capability of the population;
2. Synchronous updating and spiral learning strategies are implemented that fortify the algorithm’s ability to sidestep local optima. This approach also fosters a more efficient balance between exploitation and exploration;
3. PECSO algorithm exhibits superior global search capability, faster convergence speed and higher accuracy, as confirmed by the CEC2017 benchmark function;
4. The exceptional performance of the PECSO algorithm is further substantiated by its successful application to two practical problems.

The rest of this paper is organized as follows: Section 2 explains the foundational principles of the CSO algorithm. Section 3 introduces our proposed PECSO algorithm and elaborates on its various facets. In Section 4, we conduct benchmark function experiments using the PECSO algorithm. Section 5 demonstrates the resolution of two practical problems employing the PECSO algorithm. Finally, in Section 6, we provide a comprehensive summary of the paper, discuss the study’s limitations, and suggest directions for future research.

## 2. Chicken Swarm Optimization Algorithm

The classical CSO algorithm regards the solution of the problem as a source of food for chickens, and the fitness value in the algorithm represents the quality of the food. According to the fitness value, individuals in the chicken flock are sorted, and the flock is divided into several subgroups. Each subgroup divides the individuals into three levels: roosters, hens and chicks, and the proportions of roosters, hens and chicks are *N_r_*, *N_h_* and *N_c_*, respectively.

In the algorithm, $x_{i,j}^{t}$ represents the position of the *i*-th chicken in the *t*-th iteration of the *j*-dimensional search space. The individuals with the lowest fitness value are selected as the roosters. The roosters walk randomly in the search space, and their position is updated, as shown in Equation (1).

(1) $$x_{i,j}^{t+1}=x_{i,j}^{t}\;*\;\left(1+randn\left(0,\sigma^{2}\right)\right)\;$$

where $randn\;\left(0,\sigma^{2}\right)$ is a Gaussian distribution random number. The calculation of $\sigma^{2}$ is shown in Equation (2).

(2) $$\sigma^{2}=\left\{\begin{array}{c} \begin{array}{cc} 1, & f_{i}\leq f_{k} \end{array}\\ exp\left(\left(f_{k}-f_{i}\right)/\left|f_{i}+\varepsilon \right|\right),\;\;otherwise \end{array}\right.$$

Individuals with better fitness are selected as hens, which move following the rooster. The hen’s position is updated as shown in Equation (3).

(3) $$x_{i,j}^{t+1}=x_{i,j}^{t}+S_{1}\;*\;rand\;*\;\left(x_{r1,j}^{t}-x_{i,j}^{t}\right)+S_{2}\;*\;rand\;*\;\left(x_{r2,j}^{t}-x_{i,j}^{t}\right)$$

where *r*1 is the individual rooster followed by the *i*-th hen. The *r*2 is a randomly selected rooster or hen (*r*2 ≠ *r*1). Calculate the weights $S_{1}=exp\left((f_{i}-f_{r1})/\left(abs\left(f_{i}\right)+\varepsilon \right)\right)$ and $S_{2}=exp\left(f_{r2}-f_{i}\right)$. $f_{r1}$ and $f_{r2}$ are fitness values corresponding to *r*1 and *r*2, respectively.

Except for the roosters and hens, other individuals are defined as chicks. The chicks follow their mother’s movement, and the chick’s position is updated, as shown in Equation (4).

(4) $$x_{i,j}^{t+1}=x_{i,j}^{t}+FL\;*\;\left(x_{m,j}^{t}-x_{i,j}^{t}\right)$$

where *FL* ∈ [0, 2], $x_{m,j}^{t}$ is the position of the *i*-th mother chick.

## 3. Improved CSO Algorithm

Many variants of the CSO algorithm have been proposed. However, slow convergence speed and falling into local optimization are still the main shortcomings of the CSO algorithm in solving practical optimization problems. Therefore, to improve the convergence accuracy and speed of the CSO algorithm, a better balance between exploitation and exploration has been achieved. In this paper, we propose the PECSO algorithm, which is based on a free grouping mechanism, synchronous update and spiral learning strategies. We present the PECSO algorithm in detail, give the mathematical model and pseudocode of the PECSO algorithm, and perform a time complexity analysis.

### 3.1. New Population Distribution

**Figure 1:**
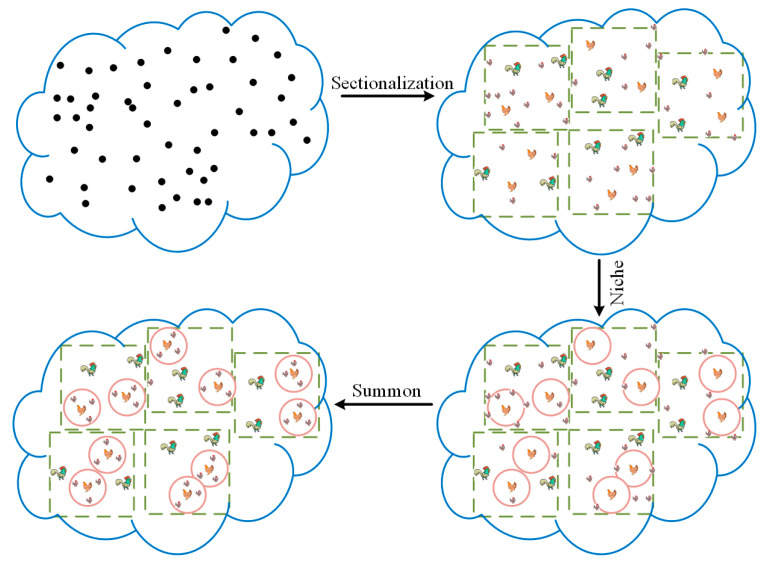
Hierarchy distribution strategy based on the free grouping mechanism.

The hierarchy structure of the CSO algorithm is established by the fitness value, which is simple but suffers from the disadvantage of the low diversity of individuals in the hierarchy. Therefore, we introduced a free grouping mechanism to redesign the swarm hierarchy, which improves the diversity of individuals in different hierarchies of the algorithm. Firstly, the method freely divides the randomly initialized population into 0.5*N_r_* groups. Within each group, roosters (*n_r_*), hens (*n_h_*) and chicks (*n_c_*) are selected based on the size of the fitness value. Secondly, multiple niches are established within the group, with the hens as the center and *L* as the radius, as shown in Equation (5). Finally, the hen summons her chicks within the niche, as shown in Equation (6). The population distribution state is formed as shown in Figure 1.

(5) $$L=\alpha \left(ub_{d}-lb_{d}\right)/N_{h}$$

(6) $$x_{c}=x_{h}+\left(2rand-1\right)\;*\;L$$

where $ub_{d}$/$lb_{d}$ is the upper/lower boundary of the *D*-dimensional solution space. α is the radius factor of the niche.

### 3.2. Individual Updating Methods

This subsection introduces several updating methods that we propose, including a best-guided search for roosters, a bi-objective search for hens and a simultaneous and spiral search for chicks.

#### 3.2.1. Best-Guided Search for Roosters

Roosters are the leaders carrying the excellent message, and their selection and updating is important. Therefore, this paper proposes the best-guided search method for roosters. Specifically, we discarded the practice of selecting a single rooster in the traditional CSO algorithm and instead selected multiple individuals with better fitness values within the group as roosters. Meanwhile, the exploration is carried out with the goal of the global optimal individual ($x_{best}$). The improved roosters can effectively utilize the historical experience of the population and have a stronger exploitation ability to overcome the problem of low convergence accuracy of the CSO algorithm. The updating step of the roosters is shown in Equation (7).

(7) $$S_{R}=x_{best}-x_{i,r}^{t}\;$$

The updated position of the roosters is shown in Equation (8).

(8) $$x_{i,r}^{t+1}=x_{i,r}^{t}+randn\left(0,\;\sigma^{2}\right)S_{R}\;$$

#### 3.2.2. Bi-Objective Search for Hens

Hens are the middle level of the CSO population and should have both exploration and exploitation capabilities and coordinate the roles of both. On the one hand, they can inherit the excellent information of the rooster. On the other hand, they repel other hens and protect the chicks from being disturbed while performing their exploratory functions. On this basis, this paper reconsiders the search goal of hens and proposes a bi-objective search strategy. Specifically, (1) combining the current optimal solution position with the position of the optimal rooster *r*1 in the group realizes the full utilization of the optimal information and improves the exploitation ability of the hen. (2) Combining the position of rooster *r*2 within the group (*r*1 ≠ *r*2) with the hen positions within other niches. It enhances diversity and enables large-scale exploration. The updating step ($S_{L}$) of the hens is shown in Equation (9).

(9) $$S_{L}=\left\{\begin{array}{cc} c_{1}\left(x_{i,r1}^{t}-x_{i,h}^{t}\right)+c_{2}\left(x_{best}-x_{i,h}^{t}\right) & if\;p<0.9\\ c_{3}\left(x_{i,r2}^{t}-x_{i,h}^{t}\right)+c_{4}\left(x_{i,k}^{t}-x_{i,h}^{t}\right) & else \end{array}\right.$$

The updated position of the hens is shown in Equation (10).

(10) $$x_{i,h}^{t+1}=x_{i,h}^{t}+\eta S_{L}$$

where *p* ∈ [0, 1]. $c_{1}$, $c_{2}$, $c_{3}$, $c_{4}$ are random numbers between 0 and 1. $x_{i,r1}^{t}$ is a rooster indexed by a hen, $x_{i,r2}^{t}$ is a randomly selected rooster (*r*2 ≠ *r*1), and $x_{i,k}^{t}$ is a competing hen (*k* ≠ *h*). $x_{i,r1}^{t}$ and $x_{i,h}^{t}$ are the position of the *i*-th rooster and hen at the *t*-th iteration, respectively. η∈ (0, 1) is the moving step factor of the niche.

#### 3.2.3. Simultaneous and Spiral Search for Chicks

**Figure 2:**
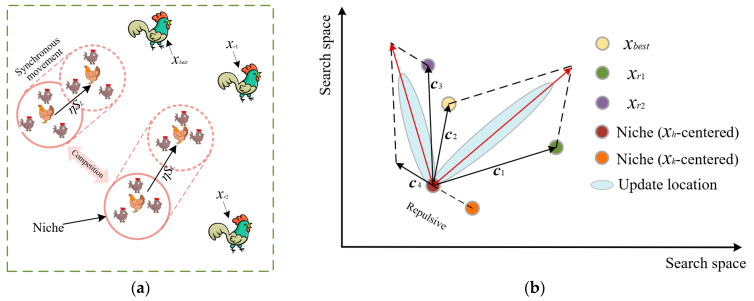
Synchronous update process of individuals in the niche. (**a**) Synchronous update trajectory. (**b**) Synchronous update of the navigation map.

**Figure 3:**
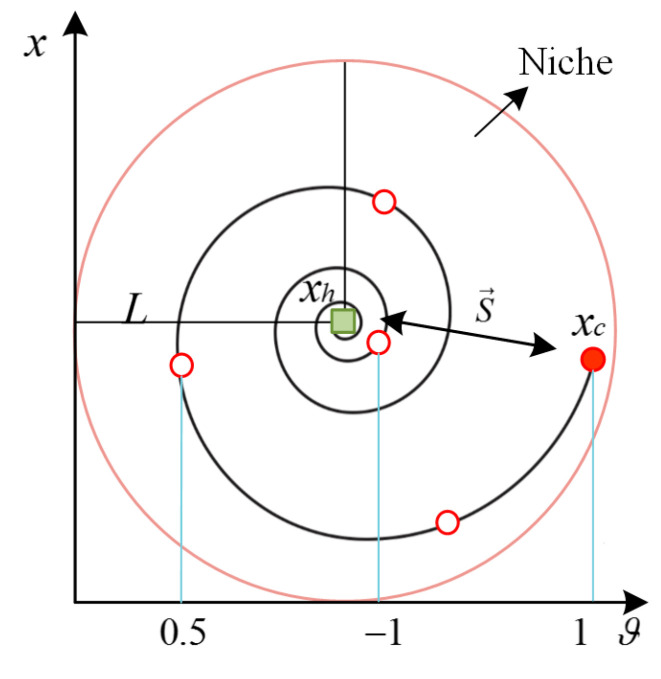
Chick’s spiral update position.

The chicks are followers of the hen and develop excellent exploration abilities by observing and learning the exploration behavior of the hen. Based on this, we propose synchronous updating and spiral learning strategies for chicks. Synchronized updating means that all chicks follow the same direction and step size as the hen for updating movement within a niche. This method can ensure the consistency of the hen and the chicks in the niche, which enhances the local exploration ability and jointly explores the potential solution space. The process of synchronous update is shown in Figure 2. Spiral learning means that individual chicks can move towards the hen (central point) in the niche, and the step size is gradually reduced during the movement, thus searching for the optimum more accurately. Specifically, the distance between the current individual chick and the hen is calculated, and the spiral radius is determined according to certain rules. Afterwards, the chick’s position is updated through the spiral radius and angle increments, and the movement trajectory is shown in Figure 3. Compared with the traditional linear updating method, the spiral update can expand the updating dimension of the chick’s position, make it more diverse and enhance the exploration ability of the algorithm. Through the synergy of synchronous updating and spiral learning, we can obtain the spiral updating step of the chicks, as shown in Equation (11).

(11) $$S_{O}=\vec{S}\;*\;e^{\beta \vartheta }\;*\;cos\left(2\pi \vartheta \right)+x_{i,h}^{t}$$

The updated position of the chicks is shown in Equation (12).

(12) $$x_{i,c}^{t+1}=x_{i,c}^{t}+\eta S_{L}+\varphi S_{O}$$

where $\vec{S}=\left|x_{i,c}^{t}-x_{i,h}^{t}\right|$, β = 1 is the logarithmic helix coefficient, and $\vartheta \in \left[-1,\;1\right]$, φ∈ (0, 1) is a random number.

### 3.3. The Implementation and Computational Complexity of PECSO Algorithm

#### 3.3.1. The Implementation of PECSO Algorithm

**Figure 4:**
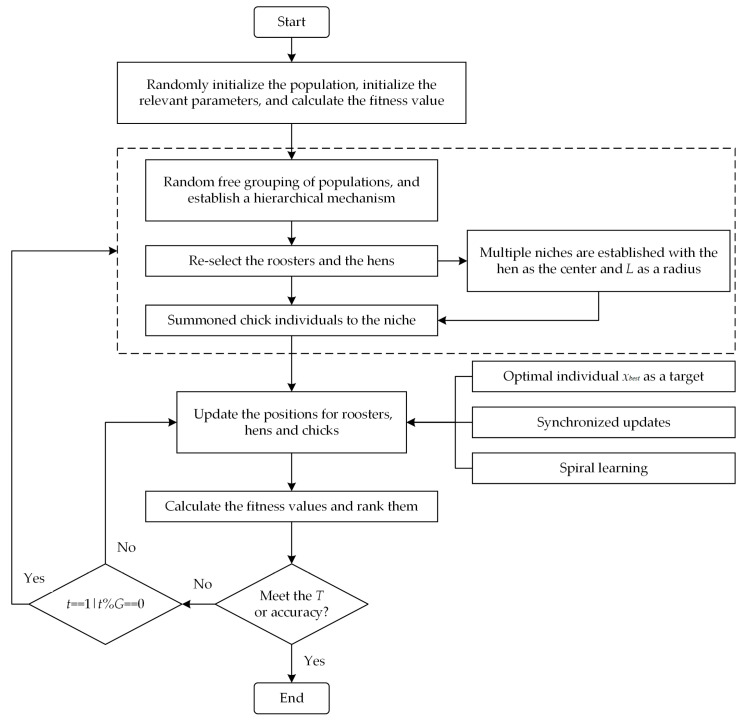
Flowchart of the PECSO algorithm.

The PECSO algorithm is used to optimize the diversity and update methods of individuals in different levels of the CSO algorithm. The specific pseudocode is given in Algorithm 1, and Figure 4 shows the flowchart of the PECSO algorithm.
**Algorithm 1:** Pseudocode of PECSO algorithmInitialize a population of *N* chickens and define the related parameters; 
While *t* < *G_max_*
 If (*t* % *G* == 0)
  Free grouping of populations and selection of roosters and hens within each group based on fitness values;
  Many niches are established with the hens as the center and *L* as the radius, according to Equations (5) and (6);
  Chicks are summoned by hens within the niche to recreate the hierarchy mechanism and to mark them.
 End if
 For *i* = 1: *N_r_*
  Update the position of the roosters by Equation (8);
 End for
 For *i* = 1: *N_h_*
  Synchronous update step of the niche is calculated by Equation (9);
  Update the position of the hens by Equation (10);
 End for
 For *i* = 1: *N_c_*
  Spiral learning of chicks by Equation (11); 
  Update the position of the chicks by Equation (12);
 End for
Evaluate the new solution, and update them if they are superior to the previous ones;
End while

#### 3.3.2. The Computational Complexity of PECSO Algorithm

The computational complexity refers to the amount of computational work required during the algorithm’s execution. It mainly depends on the number of problems executed repeatedly. The computational complexity of the PECSO algorithm is described by BigO notation. According to Algorithm 1, the population size, maximum number of iterations and dimension are represented by *N*, *T* and *D*, respectively. 

The computational complexity of the CSO algorithm mainly includes population initialization $O\left(2N\times D\right)$, population update $O\left(2N\times T\times D\right)$ and regime update $O\left(N\times T\times D/G\right)$. Therefore, $O\left(\mathrm{CSO}\right)$ is shown in Equation (13).

(13) $$O\left(\mathrm{CSO}\right)=O\left(2N\times D\right)+O\left(2N\times T\times D\right)+O\left(N\times T\times D/G\right)\;$$

The computational complexity of the PECSO algorithm mainly includes the population initialization $O\left(2N\times D\right)$, the position update of individuals within the population $O\left(2N\times T\times D\right)$, and the establishment of hierarchy $O\left(\left(N+N+N_{h}\right)\times T\times D/G\right)$ based on the free grouping strategies (messing up the order, free grouping, summoning chicks). Therefore, $O\left(\mathrm{PECSO}\right)$ is shown in Equation (14).

(14) $$O\left(\mathrm{PECSO}\right)=O\left(2N\times D\right)+O\left(2N\times T\times D\right)+O\left(\left(N+N+N_{h}\right)\times T\times D/G\right)\;$$

It can be seen from Equations (13) and (14) that the computational complexity of the PECSO and CSO algorithms is of the same order of magnitude. However, the PECSO algorithm adds two steps in updating the hierarchical relationship every G time, including the disruption of the population order and the summoning of the chicks by the hens. Therefore, the computational complexity of the PECSO algorithm is slightly higher than the CSO algorithm.

## 4. Simulation Experiment and Result Analysis

In this section, first, we perform the experimental settings, including the selection of parameters and benchmark functions. Secondly, the qualitative analysis of the PECSO algorithm is carried out in terms of four indexes (2D search history, 1D trajectory, average fitness values and convergence curves). Finally, the computational performance of the PECSO algorithm is quantitatively analyzed and compared with the other seven algorithms, in which three measurement criteria, including mean, standard deviation (std) and time, are considered; the unit of time is seconds (s).

### 4.1. Experimental Settings

Parameters: the common parameters of all algorithms are set to the same, where *N* = 100, *T* = 500, *D* = 10, 30, 50. All common parameters of the CSO algorithm include $N_{r}=0.2$, $N_{h}=0.2N$, $N_{c}=N-N_{r}-N_{h}$, G=10. Other main parameters of the algorithm are shown in Table 1. In addition, the experiment of each benchmark function is repeated 50 times to reduce the influence of random factors.

Benchmark Function: this paper selects the CEC2017 benchmark function for experiments (excluding F2) [36]. The unimodal functions (F1, F3) have only one extreme point in the search space, and it is difficult to converge to the global optimum. Therefore, the unimodal function is used to test the search accuracy. The multimodal functions (F4–F10) have multiple local extreme points, which can be used to test the global search performance. The hybrid functions (F11–F20) and composition functions (F21–F30) are a combination of unimodal and multimodal functions. More complex functions can further test the algorithm’s ability to balance exploration and exploitation.

### 4.2. Qualitative Analysis

**Figure 5:**
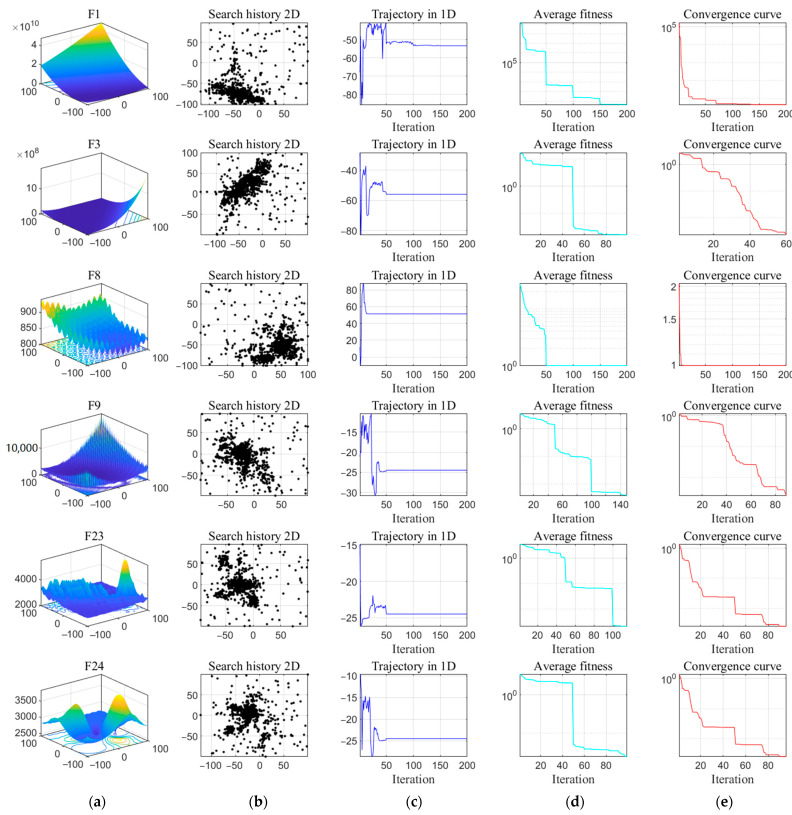
Qualitative results of PECSO algorithm: (**a**) visual diagram; (**b**) search history; (**c**) trajectory; (**d**) average fitness; (**e**) convergence curve.

The qualitative results of the PECSO algorithm are given in Figure 5, including the visualization of the benchmark function, the search history of the PECSO algorithm on the 2D benchmark test problem, the first-dimensional trajectory, the average fitness and the convergence curve. The discussion is as follows.

The second column of search history shows the location history information of each individual in the search space. It can be seen that the individuals are sparsely distributed in the search space, mostly clustered around the global optimal solution. This indicates that individuals reasonably cover a large area of the search space, and the PECSO algorithm has the exploration and exploitation abilities. However, the search history cannot show the exploratory order of individuals during the iterative process. Therefore, the third column gives the first-dimensional trajectory curves of representative individuals in each iteration. It shows the mutation of the individual during the initial iteration, which is gradually weakened throughout the iterations. According to references [37,38], this behavior ensures that the PECSO algorithm eventually converges to one point of the search space. The average fitness values and convergence curves are given in the fourth and fifth columns; it is known that the PECSO algorithm gradually approaches the optimal solution in the iterative process. Multiple convergence stages indicate that the PECSO algorithm can jump out of the local optimal value and search again, which shows that the PECSO algorithm has good local optimal avoidance ability and strong convergence ability. In brief, the PECSO algorithm effectively maintains a balance between exploitation and exploration, exhibiting advantages such as rapid convergence speed and robust global optimization capability.

### 4.3. Quantitative Analysis

This section compares the PECSO algorithm with the other seven algorithms, including PSO, CSO, MFO, WOA, and BRO, as well as the ICSO algorithm (ICSO1) proposed by Liang [29] and the improved CSO algorithm (ICSO2) proposed by Li [39], through the fitness value evaluation. The results are shown in Table 2, Table 3, Table 4, Table 5, Table 6 and Table 7, and we can draw the following conclusions.

**Figure 6:**
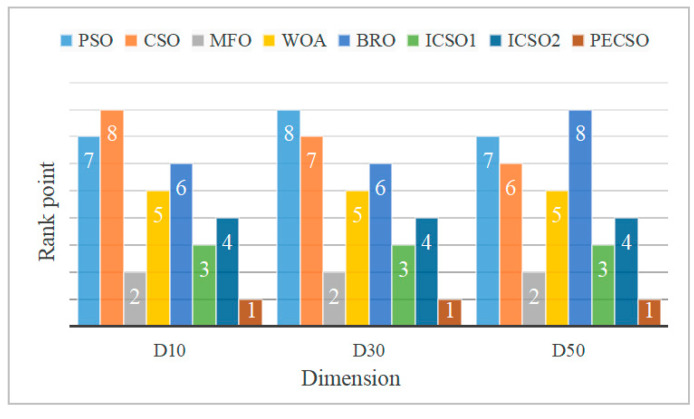
Average ranking of 8 algorithms in different dimensions.

1. From the unimodal and multimodal functions, we can find that the PECSO algorithm achieves the minimum mean and standard deviation. From the hybrid and composition functions, the PECSO algorithm obtained the best value of 80%. This shows that the PECSO algorithm has high convergence accuracy and strong global exploration ability, and its computing performance is more competitive;
2. The experimental results show that the solving ability of unimodal and multimodal functions is not affected by dimensional changes, while hybrid and composite functions get more excellent computational results in higher dimensions. This indicates that the PECSO algorithm can balance the exploitation and exploration well and has a strong ability to jump out of the local optimum. The possible reason is that the free grouping mechanism improves the establishment of the hierarchy and increases the diversity of roosters in the population. Meanwhile, synchronous updating of individuals in niche and spiral learning of chicks can effectively improve the exploitation breadth and exploration depth of the PECSO algorithm;
3. The running time of the PECSO algorithm is slightly higher than that of the CSO algorithm, but they have the same order of magnitude. It shows that the PECSO algorithm effectively improves computational performance;
4. We rank the test results of all algorithms on the benchmark function, and the average value is the indicator. Figure 6 finds that the convergence results of the PECSO algorithm are outstanding in different test dimensions.

**Figure 7:**
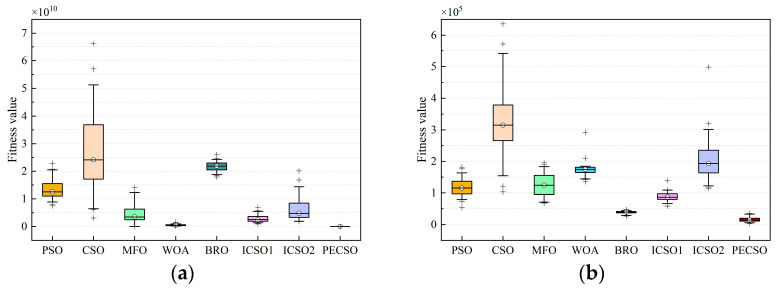

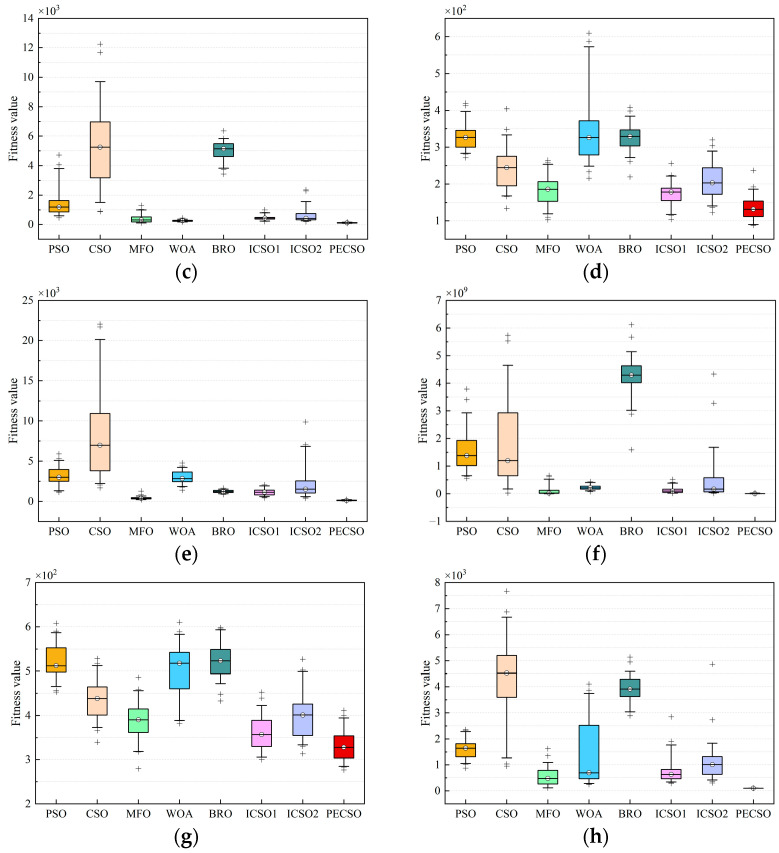
Box plots of 8 algorithms on some benchmark functions, the functions are selected as (**a**) F1; (**b**) F3; (**c**) F4; (**d**) F5; (**e**) F11; (**f**) F12; (**g**) F21; (**h**) F22.

The box plots of the eight algorithms for some benchmark functions (*D* = 30, independent experiments 50 times) are given in Figure 7. The solid line in the middle of each box represents the median fitness value, and the shorter the box and whiskers, the more concentrated the convergence results. It can be seen that the PECSO algorithm has strong stability.

**Figure 8:**
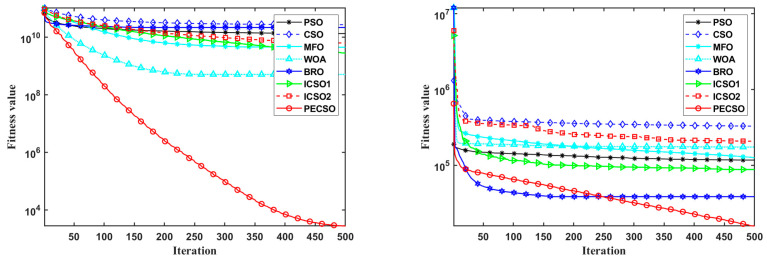

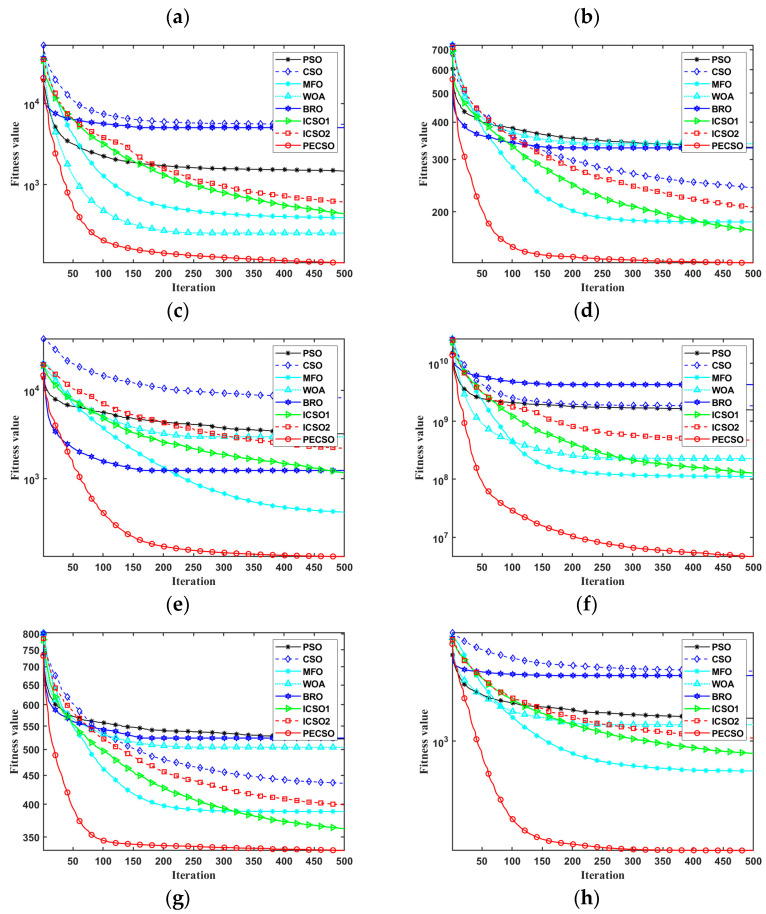
Average convergence results of 8 algorithms on benchmark functions, the functions are selected as (**a**) F1; (**b**) F3; (**c**) F4; (**d**) F5; (**e**) F11; (**f**) F12; (**g**) F21; (**h**) F22.

Figure 8 shows the convergence curves on some benchmark functions in the case of *D* = 30. Figure 8a–d shows the convergence curves of the PECSO algorithm on some unimodal and multimodal functions, which achieve the best convergence accuracy and speed. The convergence curves of the PECSO algorithm on some hybrid and composite functions are given in Figure 8f–h. In fact, there are 20 functions of this type, and the PECSO algorithm has achieved the best convergence effect on 17 benchmark functions, ranking second on three functions (F20, F23, F26). This further demonstrates the excellent convergence capability of the PECSO algorithm.

## 5. Case Analysis of Practical Application Problems

In this section, we further investigate the performance of the PECSO algorithm by solving three classical engineering optimization problems and robot inverse kinematics. Moreover, compared with other algorithms reported in the literature.

### 5.1. Engineering Optimization Problems

**Figure 9:**
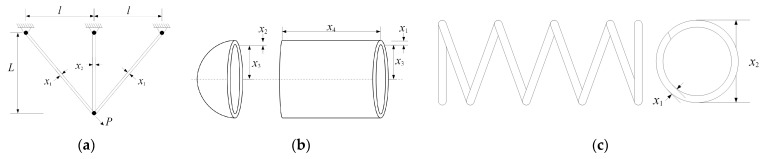
The schematic of three engineering optimization problems: (**a**) three-bar truss; (**b**) pressure vessel; (**c**) tension/compression spring.

This section selects three classical engineering application problems to test the computational potential of the PECSO algorithm in dealing with practical problems. This mainly includes the three-bar truss design [40], pressure vessel design [41] and tension/compression spring design [42]; the constraint design can be regarded as the optimal solution of the function F31, F32 and F33. For details, refer to Table 8. Their structures are shown in Figure 9.

Table 9 shows the statistical results of the three engineering optimization problems. The PECSO algorithm performs better than the CSO algorithm on three engineering optimization problems, and the results are compared with those of the FCSO algorithm in reference [43]. The results obtained by the PECSO algorithm are within the scope of practical applications and meet the constraint requirements. Meanwhile, the PECSO algorithm shows excellent applicability and stability in engineering optimization problems.

### 5.2. Solve Inverse Kinematics of PUMA 560 Robot

In this section, the PECSO algorithm is used to solve the inverse kinematics of the PUMA 560 robot, which includes the kinematics modeling, the establishment of the objective function and the simulation experiment.

#### 5.2.1. Kinematic Modeling and Objective Function Establishment

**Figure 10:**
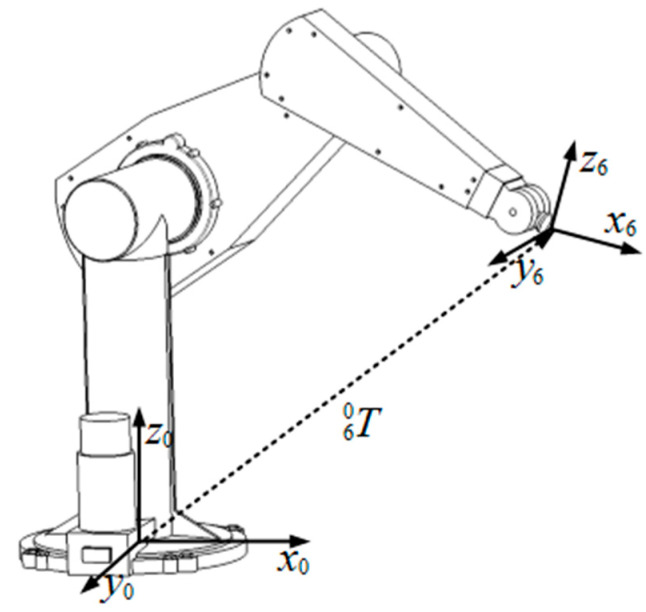
Kinematic chains solid shape of PUMA 560 robot joints.

The kinematic modeling of the robots involves establishing a coordinate system on each link of the kinematic chain, refer to Figure 10. The posture of the robot end effector is described in cartesian space by a homogeneous transformation. The kinematic equation is shown in Equation (15).

(15) T60=T10θ1·T21θ2·T32θ3·T32θ3·T65θn

The coordinate transformation relationship between adjacent links in the robot kinematic chain is obtained by the Denavit–Hartenberg (*D*-*H*) parameter method [10], as shown in Equation (16).

(16) Tii−1=cosθi−cosαi·sinθisinθicosαi·cosθisinαi·sinθiai·cosθicosθi·sinαiai·sinθi0  sinαi0   0cosαi  di0  1

The positive kinematics equation of the PUMA 560 robot can be obtained by the combination of Equations (15) and (16), as shown in Equation (17) [44].

(17) nxoxnyoyαxpxαypynzoz0  0αz pz01=T60

where n, o and α represent rotational elements of the pose matrix, and p represents the elements of the position vector.

The objective function of the solution is the Euclidean distance between the desired and actual end positions, as shown in Equation (18).

(18) $$Error=\|P-P^{\prime }\|$$

where $P=\left[p_{x},\;p_{y},\;p_{z}\right]$ represents the desired position, and $P^{\prime }=\left[p_{x}^{\prime },p_{y}^{\prime },p_{z}^{\prime }\right]$ represents the actual position.

Each joint variable needs to meet a different boundary constraint range, which is restricted by the mechanical principle of the robot, as shown in Equation (19).

(19) $$\theta_{i,min}\leq \theta_{i}\leq \theta_{i,max}\;i=1,2\ldots 6$$

where $\theta_{i,min}$/$\theta_{i,max}$ represents the upper/lower limits of the *i*-th joint variable, respectively. The specific values are shown in Table 10.

#### 5.2.2. Simulation Experiment and Analysis

The simulation experiment for solving inverse kinematics is carried out by the PECSO and CSO algorithms. The test point is a randomly selected end position within the range of movement of the PUMA 560 robot. The relevant parameter settings are the same as in Section 4.1, and the experimental results are shown in Table 11; among them, the results of the BRO algorithm are taken from reference [10]. The results show that the PECSO algorithm has higher solution accuracy than the CSO and BRO algorithms, which also indicates that the PECSO algorithm is feasible for solving the robot kinematic inverse. Moreover, with the increase in the *N* and the *T*, the computational performance of all algorithms is gradually enhanced. We find that the change of the *T* has a greater impact on the calculation results.

## 6. Conclusions

This paper proposes a CSO algorithm with a performance-enhanced strategy. The algorithm utilizes a free grouping mechanism to establish a hierarchy and select the roosters and hens. Establishing a niche centered around hens and gathering chicks. Roosters are updated with the goal of global optimum, and hens and chicks are updated synchronously in the niche. To increase exploration capability, chicks also perform spiral learning. They improve the singularity of rooster selection and the simplicity of individual position updating and effectively enhance the overall performance of the CSO algorithm. In the simulation, 29 benchmark functions are utilized to verify that the PECSO algorithm has outstanding performance in comparison with the other seven algorithms. In addition, three engineering optimization problems and PMUA 560 robot inverse kinematics solutions are solved based on the PECSO algorithm. It shows that the PECSO algorithm has excellent universality in complex practical problems and has certain practicability and development prospects in solving optimization problems.

The high-performance PECSO algorithm is of great significance for solving complex problems, improving search efficiency, enhancing robustness and adapting to dynamic environments. However, there are still some limitations. From the qualitative analysis, it can be found that the running time of the PECSO algorithm is slightly higher than that of the CSO algorithm. When dealing with large-scale data and complex problems, it may lead to an increase in computational complexity, and the running time of the PECSO algorithm will also increase, which may result in a large distance from the CSO algorithm in terms of running time. Next, to obtain better results, we can focus our main research direction on reducing the computational complexity of the algorithms. 

## Figures and Tables

**Table 1:** The main parameters of the 8 algorithms.

Algorithms	Parameters
PSO	The inertia weight is *w* = 0.8, the two learning factors are *c_1_* = *c_2_* = 2, *V_max_* = 1.5, *V_min_* = −1.5
CSO	*FL* ∈ [0, 2]
MFO	*b* = 1, *t* = [−1, 1], *a* ∈ [−1, −2]
WOA	*a* is decreasing linearly from 2 to 0, *b* = 1
BRO	Maximum damage is 3
ICSO1	*FL* ∈ [0.4, 1], *c* = 10, *λ* = 1.5
ICSO2	*FL* ∈ [0.4, 0.9], *ω_max_* = 0.9, *ω_min_* = 0.4, *K* = 200
PECSO	*η* = 0.5, *α* = 1

**Table 2:** Results and comparison of all algorithms on unimodal and multimodal functions (*D* = 10).

Func.	Index	PSO	CSO	MFO	WOA	BRO	ICSO1	ICSO2	PECSO
F1	Mean	1.19 × 10^9^	2.08 × 10^9^	7.77 × 10^3^	8.84 × 10^6^	1.7 × 10^9^	3.44 × 10^6^	6.76 × 10^7^	1205.033
	Std	8.40 × 10^8^	1.81 × 10^9^	5.18 × 10^3^	7.28 × 10^6^	3.5 × 10^8^	1.03 × 10^7^	2.74 × 10^8^	1358.712
	Time	0.13	0.27	0.20	0.17	2.24	0.87	0.28	0.43
F3	Mean	7607.653	1.65 × 10^4^	4505.391	1505.661	1214.764	2539.529	9645.902	0.00211
	Std	4050.991	8819.233	5588.509	962.3674	374.3617	1392.153	8211.638	0.00220
	Time	0.13	0.26	0.20	0.17	2.48	0.85	0.27	0.41
F4	Mean	64.25915	105.1704	12.03310	41.85066	117.9152	16.23396	35.22256	3.47833
	Std	33.79702	118.5975	16.94665	29.63701	33.33364	24.35405	36.09982	2.21821
	Time	0.13	0.25	0.20	0.16	2.61	0.85	0.27	0.41
F5	Mean	51.23975	36.93607	28.44677	40.73687	49.00566	25.46498	31.51915	14.01220
	Std	7.510360	15.42578	10.77455	9.579730	9.96857	9.699020	12.83864	6.215580
	Time	0.15	0.27	0.22	0.18	2.54	0.85	0.29	0.43
F6	Mean	21.27100	3.531760	0.522634	38.14514	28.20401	2.464216	6.380108	0.011514
	Std	5.764562	4.680841	1.625069	12.80828	6.99729	2.294613	6.776781	0.077628
	Time	0.21	0.34	0.28	0.25	2.66	0.93	0.35	0.50
F7	Mean	94.9378	40.82997	34.41994	68.27110	50.69352	37.71385	35.06131	27.01692
	Std	13.70433	11.94847	13.11673	15.43730	12.08821	10.29097	9.679454	7.839129
	Time	0.16	0.27	0.23	0.20	2.56	0.86	0.30	0.44
F8	Mean	54.88400	25.66856	26.53715	55.4255	33.15502	24.21200	28.89014	15.50151
	Std	7.907872	11.31825	10.50200	16.73376	10.29395	9.128454	12.48659	4.807887
	Time	0.15	0.28	0.22	0.19	2.53	0.86	0.29	0.43
F9	Mean	248.4728	325.4400	7.10053	761.833	201.9952	54.94315	169.3408	0.009137
	Std	133.3334	323.3972	28.2465	336.279	117.3242	97.29246	263.7057	0.064244
	Time	0.16	0.27	0.23	0.19	2.47	0.86	0.30	0.43
F10	Mean	1145.507	1080.774	868.3683	832.6663	1137.677	947.9620	1008.287	580.5303
	Std	235.2473	368.7106	278.4062	297.1957	263.6751	306.0519	314.4698	227.9308
	Time	0.17	0.28	0.24	0.20	2.57	0.87	0.31	0.44

**Table 3:** Results and comparison of all algorithms on hybrid and composition functions (*D* = 10).

Func.	Index	PSO	CSO	MFO	WOA	BRO	ICSO1	ICSO2	PECSO
F11	Mean	311.2918	278.9675	28.77234	159.7373	90.95789	65.08224	184.2798	22.71487
	Std	142.0046	417.4833	43.16772	75.32435	26.06289	51.13908	187.4143	13.87131
	Time	0.14	0.26	0.22	0.18	2.49	0.86	0.28	0.42
F12	Mean	7.32 × 10^6^	5.06 × 10^7^	1.16 × 10^6^	2.99 × 10^6^	1.06 × 10^6^	3.73 × 10^6^	5.73 × 10^6^	2.25 × 10^4^
	Std	5.24 × 10^6^	1.47 × 10^8^	3.51 × 10^6^	3.08 × 10^6^	6.33 × 10^5^	5.43 × 10^6^	6.98 × 10^6^	1.76 × 10^4^
	Time	0.14	0.27	0.22	0.18	2.61	0.86	0.28	0.42
F13	Mean	1.30 × 10^5^	2.08 × 10^4^	9173.812	3.78 × 10^4^	2.39 × 10^4^	1.69 × 10^4^	2.87 × 10^4^	1.22 × 10^4^
	Std	8.75 × 10^4^	1.41 × 10^4^	1.04 × 10^4^	4420.481	6179.173	1.14 × 10^4^	2.55 × 10^4^	7825.147
	Time	0.15	0.27	0.22	0.19	2.74	0.86	0.28	0.43
F14	Mean	237.0072	1915.462	886.1556	360.5561	748.7817	394.2500	830.6854	208.7593
	Std	114.1593	1134.785	992.0691	534.7172	841.1497	532.2103	829.9934	225.2404
	Time	0.16	0.28	0.24	0.20	2.72	0.88	0.30	0.44
F15	Mean	1.08 × 10^4^	1.51 × 10^4^	3901.560	2445.835	5238.864	2894.893	1.79 × 10^4^	635.7582
	Std	1.26 × 10^4^	2.37 × 10^4^	4555.720	1718.992	3064.849	4904.754	2.58 × 10^4^	898.4517
	Time	0.14	0.26	0.21	0.18	2.77	0.85	0.27	0.42
F16	Mean	95.86476	246.3651	96.77121	76.65239	221.5054	127.2405	189.5364	128.5848
	Std	39.74258	193.3885	101.9082	60.69622	94.56331	96.35577	162.7510	133.6224
	Time	0.15	0.28	0.22	0.20	2.81	0.87	0.29	0.43
F17	Mean	115.8047	78.98954	39.90501	100.6452	79.91754	51.40780	75.76799	34.72186
	Std	22.39313	59.79084	17.90421	23.62764	14.18924	20.63777	47.81470	18.89641
	Time	0.20	0.32	0.28	0.24	2.79	0.92	0.35	0.49
F18	Mean	9.44 × 10^4^	1.38 × 10^4^	1.98 × 10^4^	3677.271	2341.611	2.07 × 10^4^	1.63 × 10^4^	4234.677
	Std	8.17 × 10^4^	1.41 × 10^4^	1.37 × 10^4^	5591.377	3629.037	1.80 × 10^4^	1.40 × 10^4^	4720.534
	Time	0.15	0.27	0.23	0.19	2.66	0.87	0.29	0.43
F19	Mean	3185.175	1.57 × 10^4^	3953.265	3.59 × 10^4^	5630.787	3064.998	2.55 × 10^4^	2063.317
	Std	4483.488	3.58 × 10^4^	6371.632	1.72 × 10^4^	3265.784	4810.707	4.74 × 10^4^	1732.792
	Time	0.47	0.55	0.55	0.51	3.06	1.19	0.61	0.75
F20	Mean	114.1371	81.40005	40.45046	163.3919	115.7927	69.73579	71.57455	31.83129
	Std	26.52308	68.79990	26.45577	68.67892	48.15426	55.64153	47.16772	23.36321
	Time	0.21	0.33	0.28	0.25	2.68	0.93	0.35	0.50
F21	Mean	108.5544	175.3791	179.7778	113.0315	121.3235	116.6314	132.7406	124.6529
	Std	3.916455	69.59982	61.90168	18.75488	7.038273	25.59729	35.70672	49.31258
	Time	0.21	0.33	0.28	0.25	2.60	0.93	0.34	0.49
F22	Mean	160.7779	189.0434	103.9039	124.7609	179.4140	103.5302	146.9429	103.5816
	Std	25.27810	181.7540	11.49952	19.32559	27.03703	27.94584	80.23289	13.24524
	Time	0.24	0.33	0.32	0.28	2.67	0.94	0.39	0.52
F23	Mean	354.0431	335.8440	323.8059	347.7348	378.6894	327.7684	327.9389	319.8174
	Std	14.31813	13.69908	7.757381	13.54920	37.65398	10.78876	11.14153	7.389369
	Time	0.26	0.35	0.34	0.30	2.69	0.95	0.39	0.54
F24	Mean	390.1786	380.6473	342.3239	390.7160	278.5041	321.0273	357.7536	342.1947
	Std	12.02908	16.33176	59.20593	19.66264	135.5445	93.89438	46.84364	50.68501
	Time	0.26	0.37	0.35	0.31	2.74	0.97	0.41	0.55
F25	Mean	474.3985	488.2035	435.5922	450.2481	477.5108	438.5894	457.1487	429.4796
	Std	24.39434	58.48867	20.18933	12.66054	15.21807	23.67865	32.58971	22.76244
	Time	0.23	0.33	0.31	0.28	2.82	0.94	0.37	0.51
F26	Mean	480.2783	546.6617	391.4012	669.7582	709.4012	410.5817	440.7229	343.9639
	Std	66.33779	208.9693	29.35429	186.9457	145.4170	69.87310	102.7568	178.5598
	Time	0.29	0.37	0.36	0.33	2.82	1.00	0.43	0.57
F27	Mean	421.9835	404.2941	392.2372	400.5075	459.2579	394.4270	398.4692	378.7198
	Std	22.36179	10.36445	1.768843	6.300893	21.39002	3.466860	14.86603	2.289450
	Time	0.29	0.40	0.37	0.34	2.72	1.00	0.44	0.55
F28	Mean	629.9686	593.1006	488.41981	549.12104	542.0223	567.1627	563.77963	477.4404
	Std	48.01225	119.1607	94.850651	109.08613	114.3551	145.1582	131.39175	50.74241
	Time	0.27	0.37	0.35	0.31	2.78	0.97	0.41	0.55
F29	Mean	440.9476	420.3877	307.4976	599.8618	385.9158	346.8368	378.78393	332.0303
	Std	90.66713	78.36339	42.56096	100.5884	55.32008	68.56983	91.612929	51.85453
	Time	0.27	0.37	0.34	0.31	2.88	0.99	0.41	0.55
F30	Mean	8.99 × 10^5^	2.36 × 10^6^	6.59 × 10^5^	8.97 × 10^5^	1.06 × 10^6^	7.26 × 10^5^	1.13 × 10^6^	7980.850
	Std	3.76 × 10^5^	2.91 × 10^6^	4.25 × 10^5^	8.83 × 10^5^	7.47 × 10^5^	2.37 × 10^5^	7.85 × 10^5^	1.07 × 10^4^
	Time	0.53	0.62	0.62	0.58	2.98	1.26	0.67	0.82

**Table 4:** Results and comparison of all algorithms on unimodal and multimodal functions (*D* = 30).

Func.	Index	PSO	CSO	MFO	WOA	BRO	ICSO1	ICSO2	PECSO
F1	Mean	1.34 × 10^10^	2.73 × 10^10^	4.483 × 10^9^	5.09 × 10^8^	2.17 × 10^10^	2.81 × 10^9^	6.261 × 10^9^	2739.501
	Std	3.48 × 10^9^	1.52 × 10^10^	3.413 × 10^9^	2.78 × 10^8^	1.80 × 10^9^	1.23 × 10^9^	4.036 × 10^9^	3625.805
	Time	0.95	0.44	0.34	3.62	0.65	2.42	0.70	0.58
F3	Mean	1.17 × 10^5^	3.28 × 10^5^	1.25 × 10^5^	1.74 × 10^5^	3.85 × 10^4^	8.77 × 10^4^	2.07 × 10^5^	1.58 × 10^4^
	Std	2.71 × 10^4^	1.09 × 10^5^	3.50 × 10^4^	2.16 × 10^4^	4410.073	1.44 × 10^4^	6.49 × 10^5^	7661.569
	Time	0.35	0.66	0.59	0.45	3.56	2.43	0.70	0.92
F4	Mean	1469.524	5544.343	390.5003	252.0710	5043.692	436.8082	609.4365	108.1792
	Std	914.9974	3144.406	275.1218	53.93749	607.4736	147.1840	483.4547	24.44122
	Time	0.34	0.65	0.57	0.44	3.54	2.42	0.70	0.90
F5	Mean	329.0702	241.3721	184.9542	338.5593	327.8808	173.2287	206.7531	134.8208
	Std	34.11802	51.75799	41.84081	83.83817	35.88941	29.59733	44.61135	30.73982
	Time	0.41	0.69	0.64	0.51	3.67	2.43	0.76	0.99
F6	Mean	58.26297	24.77571	28.89898	75.02079	71.26934	23.03547	29.41769	15.95344
	Std	8.937212	8.318971	12.99185	7.375103	6.249166	6.259538	7.648161	5.371144
	Time	0.62	0.93	0.86	0.71	3.82	2.69	0.98	1.20
F7	Mean	605.8144	566.8085	270.0943	496.9897	462.7574	305.4317	411.9734	236.5616
	Std	92.37522	233.9484	94.55899	74.62911	69.88441	56.01690	146.0721	61.23752
	Time	0.44	0.72	0.66	0.53	3.69	2.49	0.79	1.00
F8	Mean	321.0630	204.4143	188.0338	217.3065	268.7952	159.4270	189.6993	112.6484
	Std	29.96028	44.23809	45.15304	51.75257	30.97851	27.43132	43.25428	21.42594
	Time	0.42	0.70	0.65	0.52	3.65	2.47	0.77	1.00
F9	Mean	8833.940	7870.299	5318.818	8419.494	7220.843	3734.741	6789.744	2230.670
	Std	2717.427	1822.299	1927.105	2392.576	1745.162	1155.832	2678.077	672.1063
	Time	0.43	0.71	0.65	0.52	3.70	2.50	0.77	1.00
F10	Mean	7268.225	5052.111	4223.846	5851.981	7032.766	4593.454	4732.035	3558.053
	Std	579.4093	1015.415	601.8196	624.8404	552.8582	1112.350	1007.524	512.6436
	Time	0.47	0.73	0.70	0.56	3.67	2.51	0.83	1.04

**Table 5:** Results and comparison of all algorithms on hybrid and composition functions (*D* = 30).

Func.	Index	PSO	CSO	MFO	WOA	BRO	ICSO1	ICSO2	PECSO
F11	Mean	3203.062	8204.801	415.7548	2976.849	1235.297	1163.585	2201.071	130.4050
	Std	1116.118	5263.914	173.7117	771.5592	183.0142	424.4723	1927.311	46.74434
	Time	0.38	0.70	0.62	0.48	3.70	2.46	0.74	0.96
F12	Mean	1.56 × 10^9^	1.84 × 10^9^	1.12 × 10^8^	2.26 × 10^8^	4.27 × 10^9^	1.27 × 10^8^	4.71 × 10^8^	4.63 × 10^6^
	Std	7.45 × 10^8^	1.71 × 10^9^	1.76 × 10^8^	8.09 × 10^7^	7.23 × 10^8^	1.15 × 10^8^	7.89 × 10^8^	6.48 × 10^6^
	Time	0.42	0.73	0.66	0.52	3.65	2.51	0.78	1.00
F13	Mean	4.82 × 10^8^	6.96 × 10^8^	7.60 × 10^6^	4.94 × 10^5^	1.49 × 10^9^	7.07 × 10^6^	8.29 × 10^7^	8997.900
	Std	6.87 × 10^8^	1.32 × 10^9^	2.16 × 10^7^	4.03 × 10^5^	6.69 × 10^8^	1.71 × 10^7^	2.79 × 10^8^	7355.072
	Time	0.41	0.71	0.64	0.50	3.69	2.49	0.76	0.98
F14	Mean	1.21 × 10^6^	2.81 × 10^6^	2.22 × 10^5^	1.56 × 10^6^	4.15 × 10^5^	4.19 × 10^5^	2.06 × 10^6^	8.96 × 10^4^
	Std	8.21 × 10^5^	4.52 × 10^6^	3.02 × 10^5^	8.01 × 10^5^	1.80 × 10^5^	3.84 × 10^5^	3.40 × 10^6^	6.69 × 10^4^
	Time	0.46	0.76	0.70	0.57	3.68	2.55	0.82	1.05
F15	Mean	4.73 × 10^7^	6.83 × 10^7^	4.57 × 10^4^	2.94 × 10^5^	1.76 × 10^4^	1.19 × 10^5^	1.82 × 10^7^	2457.060
	Std	3.88 × 10^7^	2.76 × 10^8^	5.23 × 10^4^	2.75 × 10^5^	8239.138	9.38 × 10^4^	1.28 × 10^8^	2232.645
	Time	0.37	0.68	0.62	0.47	3.63	2.46	0.73	0.97
F16	Mean	2463.687	2046.790	1424.636	2526.603	2844.388	1573.644	1784.144	1024.343
	Std	321.4705	444.9287	354.0005	457.4043	534.7903	335.2769	476.9434	296.9336
	Time	0.42	0.72	0.65	0.52	3.66	2.50	0.77	0.99
F17	Mean	1244.203	1160.452	741.8861	1117.362	1097.900	888.3213	938.2913	594.9978
	Std	166.6690	310.1898	230.2786	217.6323	245.0365	216.0549	234.7641	178.2540
	Time	0.60	0.88	0.84	0.70	3.84	2.70	0.97	1.19
F18	Mean	1.10× 10^7^	2.78 × 10^7^	4.59 × 10^6^	7.79 × 10^6^	1.89 × 10^6^	4.26 × 10^6^	1.78 × 10^7^	1.24 × 10^6^
	Std	2.16 × 10^7^	4.76 × 10^7^	6.17 × 10^6^	6.71 × 10^6^	1.29 × 10^6^	5.93 × 10^6^	2.10 × 10^7^	1.22 × 10^6^
	Time	0.41	0.73	0.65	0.51	3.64	2.49	0.77	0.99
F19	Mean	9.85 × 10^7^	7.53 × 10^7^	1.16 × 10^7^	7.90 × 10^6^	3.20 × 10^6^	6.46 × 10^6^	3.63 × 10^7^	1.31 × 10^4^
	Std	7.74 × 10^7^	2.78 × 10^8^	3.77 × 10^7^	5.45 × 10^6^	1.55 × 10^6^	1.09 × 10^7^	6.26 × 10^7^	8282.171
	Time	1.49	1.66	1.73	1.59	4.72	3.58	1.85	2.08
F20	Mean	875.0725	460.3876	600.2112	730.9683	698.5802	651.5624	682.8055	589.3753
	Std	107.2989	157.7933	167.0108	135.9671	132.8971	187.7451	165.6334	154.3269
	Time	0.64	0.91	0.88	0.74	3.86	2.70	1.01	1.22
F21	Mean	521.9030	435.4149	388.0635	504.4011	523.8071	361.9451	399.0245	331.2058
	Std	37.39066	41.75662	42.00323	55.53786	38.01514	37.69702	46.96924	32.57443
	Time	0.73	1.03	0.96	0.83	3.95	2.80	1.10	1.31
F22	Mean	1613.941	4313.309	535.5631	1411.673	3937.591	771.1468	1065.725	100.899
	Std	382.6647	1549.842	339.0054	1276.843	470.3364	483.0173	716.9757	1.40774
	Time	0.81	1.01	1.04	0.90	4.03	2.83	1.18	1.38
F23	Mean	852.2221	592.1274	516.2654	733.8387	1047.188	567.1112	564.7586	517.8929
	Std	69.85124	62.10348	33.83017	88.55069	87.56876	58.05066	57.36734	34.17164
	Time	0.87	1.10	1.11	0.98	4.12	2.90	1.26	1.46
F24	Mean	975.0453	707.0991	577.1475	801.7843	1161.468	638.8340	640.0261	577.9206
	Std	63.80724	63.12925	32.179085	90.57335	74.83691	58.12701	53.00829	41.37280
	Time	0.94	1.18	1.19	1.05	4.19	2.98	1.33	1.52
F25	Mean	1506.003	1705.441	490.2608	604.7758	982.2687	606.5717	765.2691	410.8481
	Std	324.0564	946.0736	81.88208	28.95652	54.19007	79.75298	221.3287	17.81139
	Time	0.86	1.13	1.09	0.96	4.09	2.96	1.24	1.43
F26	Mean	4852.768	4253.476	2722.607	5958.751	6565.972	3327.392	3697.881	3090.922
	Std	533.5813	573.0618	274.6280	1007.016	473.8096	653.7841	651.5328	794.3931
	Time	1.05	1.25	1.29	1.14	4.33	3.13	1.44	1.63
F27	Mean	937.8946	623.9552	535.5071	678.1779	1395.561	589.2438	608.5513	500.007
	Std	106.1911	59.64512	18.59942	87.56073	151.7527	44.38437	55.08369	0.00022
	Time	1.17	1.42	1.42	1.28	4.48	3.22	1.56	1.74
F28	Mean	1692.889	2461.631	849.0510	690.7208	2101.717	728.9605	1165.746	499.0489
	Std	524.0667	1250.615	341.0864	66.65202	153.0218	113.1344	914.9074	25.70241
	Time	1.03	1.28	1.27	1.14	4.28	3.13	1.42	1.60
F29	Mean	2052.864	1743.186	1112.501	2680.039	2848.197	1492.507	1647.332	957.8104
	Std	317.6705	459.9219	239.7581	405.3932	436.8206	384.2833	373.5187	260.3517
	Time	0.89	1.15	1.13	0.99	4.14	3.01	1.27	1.45
F30	Mean	1.41 × 10^8^	4.20 × 10^7^	2.24 × 10^5^	7.23 × 10^7^	7.52 × 10^7^	7,37 × 10^6^	1.61 × 10^7^	7395.534
	Std	4.34 × 10^7^	1.76 × 10^8^	3.11 × 10^5^	3.01 × 10^7^	3.70 × 10^7^	1.13 × 10^7^	2.21 × 10^7^	8448.894
	Time	1.78	1.94	2.01	1.88	5.02	3.89	2.15	2.34

**Table 6:** Results and comparison of all algorithms on unimodal and multimodal functions (*D* = 50).

Func.	Index	PSO	CSO	MFO	WOA	BRO	ICSO1	ICSO2	PECSO
F1	Mean	5.63 × 10^5^	3.49 × 10^9^	4.41 × 10^10^	6.74 × 10^10^	7.75 × 10^10^	1.94 × 10^10^	2.66 × 10^10^	2.84 × 10^10^
	Std	1.81 × 10^5^	1.22 × 10^9^	1.2 × 10^10^	3.75 × 10^9^	2.05 × 10^10^	4.69 × 10^9^	9.15 × 10^9^	1.27 × 10^10^
	Time	0.74	0.40	0.36	2.74	0.48	1.28	0.52	0.57
F3	Mean	3.44 × 10^5^	6.02 × 10^5^	3.29 × 10^5^	1.68 × 10^5^	1.24 × 10^5^	2.06 × 10^5^	4.26 × 10^5^	1.09 × 10^5^
	Std	6.26 × 10^4^	2.17 × 10^5^	6.39 × 10^4^	1.85 × 10^4^	8174.464	3.29 × 10^4^	8.94 × 10^4^	2.13 × 10^4^
	Time	0.36	0.48	0.57	0.40	2.84	1.28	0.51	0.75
F4	Mean	5690.752	1.69 × 10^4^	2609.911	1043.799	1.83 × 10^4^	2672.436	3252.521	231.4210
	Std	2289.673	1.09 × 10^4^	1572.541	319.6070	1838.551	855.6603	2307.473	64.89824
	Time	0.38	0.50	0.61	0.43	2.94	1.36	0.55	0.80
F5	Mean	650.2707	491.5995	430.7602	452.2232	541.0584	385.6534	443.5153	244.8575
	Std	38.15368	60.02821	59.24524	72.94412	37.07843	42.89941	56.04680	36.71267
	Time	0.46	0.52	0.67	0.50	2.96	1.31	0.62	0.90
F6	Mean	78.94875	42.80420	49.25934	93.77500	88.51010	41.33403	48.07438	34.50339
	Std	11.71388	7.176038	9.031867	10.25064	7.601178	7.413033	8.657931	8.025067
	Time	0.76	0.90	0.98	0.80	3.17	1.68	0.91	1.19
F7	Mean	1463.551	1514.436	1162.377	1049.873	1114.199	774.5831	1093.967	594.6951
	Std	167.0322	410.5448	392.3836	125.5476	93.95031	92.84511	219.4935	98.13384
	Time	0.47	0.54	0.69	0.52	2.95	1.37	0.63	0.91
F8	Mean	680.3976	532.7670	426.4445	550.0613	582.9400	412.1534	454.2666	282.2703
	Std	59.53979	81.32572	62.91386	99.01249	37.21877	49.50979	55.77509	47.00482
	Time	0.48	0.54	0.70	0.53	2.85	1.34	0.64	0.94
F9	Mean	3.54 × 10^4^	3.53 × 10^4^	1.53 × 10^4^	2.79 × 10^4^	2.98 × 10^4^	1.68 × 10^4^	2.55 × 10^4^	8737.336
	Std	7605.117	7445.706	4118.053	7288.775	4430.718	3228.336	8366.852	2363.323
	Time	0.48	0.54	0.68	0.52	3.00	1.40	0.64	0.89
F10	Mean	1.29 × 10^4^	8245.27	7648.661	1.14 × 10^4^	1.31 × 10^4^	9208.548	9198.573	6289.793
	Std	939.4008	770.339	1052.331	1215.152	763.5311	1548.342	1202.687	761.1483
	Time	0.54	0.57	0.75	0.58	3.10	1.38	0.69	0.95

**Table 7:** Results and comparison of all algorithms on hybrid and composition functions (*D* = 50).

Func.	Index	PSO	CSO	MFO	WOA	BRO	ICSO1	ICSO2	PECSO
F11	Mean	1.49 × 10^4^	1.39 × 10^4^	3035.501	2262.674	9726.280	6716.607	1.08 × 10^4^	297.8482
	Std	4415.406	5694.763	2031.633	611.7686	1325.523	2346.000	3821.738	65.36438
	Time	0.41	0.51	0.63	0.45	2.91	1.34	0.56	0.84
F12	Mean	1.10 × 10^10^	2.17 × 10^10^	2.82 × 10^9^	5.86 × 10^8^	3.84 × 10^10^	1.71 × 10^9^	3.27 × 10^9^	7.60 × 10^6^
	Std	4.34 × 10^9^	1.24 × 10^10^	2.34 × 10^9^	2.45 × 10^8^	4.93 × 10^9^	7.31 × 10^8^	3.53 × 10^9^	6.38 × 10^6^
	Time	0.49	0.58	0.70	0.53	3.06	1.44	0.64	0.90
F13	Mean	1.34 × 10^10^	4.92 × 10^9^	3.03 × 10^8^	2.14 × 10^7^	1.35 × 10^10^	2.19 × 10^8^	9.31 × 10^8^	1.28 × 10^4^
	Std	7.95 × 10^9^	5.59 × 10^9^	6.37 × 10^8^	1.93 × 10^7^	3.35 × 10^9^	2.14 × 10^8^	2.25 × 10^9^	5481.711
	Time	0.43	0.53	0.65	0.48	2.89	1.36	0.58	0.86
F14	Mean	3.47 × 10^6^	1.16 × 10^7^	2.04 × 10^6^	2.09 × 10^6^	8.68 × 10^6^	4.24 × 10^6^	7.63 × 10^6^	5.87 × 10^5^
	Std	1.33 × 10^6^	2.06 × 10^7^	2.71 × 10^6^	1.19 × 10^6^	4.27 × 10^6^	3.71 × 10^6^	1.14 × 10^7^	4.96 × 10^5^
	Time	0.54	0.60	0.76	0.59	2.99	1.46	0.68	0.97
F15	Mean	9.36 × 10^8^	6.9 × 10^8^	4.82 × 10^7^	3.85 × 10^6^	1.55 × 10^9^	2.09 × 10^7^	3.59 × 10^8^	6099.017
	Std	6.20 × 10^8^	1.36 × 10^9^	1.53 × 10^8^	3.87 × 10^6^	6.37 × 10^8^	4.34 × 10^7^	7.18 × 10^8^	8801.897
	Time	0.40	0.51	0.63	0.45	2.74	1.33	0.55	0.83
F16	Mean	4411.178	3637.562	2596.259	4285.890	5069.837	2921.396	3255.263	1616.872
	Std	498.3067	650.2152	474.9358	1016.954	641.6171	542.7487	659.8195	413.2548
	Time	0.46	0.54	0.67	0.50	2.91	1.37	0.61	0.84
F17	Mean	3593.128	4266.580	2120.972	2585.764	2456.910	2309.732	2834.776	1531.586
	Std	421.8792	3966.818	526.7664	458.2317	379.7385	374.7005	699.3271	323.0368
	Time	0.72	0.76	0.93	0.76	3.21	1.66	0.87	1.13
F18	Mean	2.39 × 10^7^	3.67 × 10^7^	7.77 × 10^7^	1.54 × 10^7^	1.97 × 10^7^	1.35 × 10^7^	2.78 × 10^7^	3.63 × 10^6^
	Std	8.35 × 10^6^	3.27 × 10^7^	6.23 × 10^6^	3.79 × 10^6^	5.16 × 10^6^	1.01 × 10^7^	2.96 × 10^7^	2.03 × 10^6^
	Time	0.45	0.55	0.65	0.49	3.02	1.37	0.59	0.86
F19	Mean	2.23 × 10^8^	4.61 × 10^8^	9.93 × 10^6^	3.88 × 10^6^	5.32 × 10^8^	9.01 × 10^6^	9.22 × 10^7^	1.15 × 10^4^
	Std	7.95 × 10^7^	6.02 × 10^8^	3.53 × 10^7^	2.95 × 10^6^	2.42 × 10^8^	9.42 × 10^6^	2.69 × 10^8^	6970.648
	Time	2.06	1.95	2.28	2.10	4.62	3.00	2.22	2.48
F20	Mean	2101.191	1766.057	1504.138	1631.001	1658.922	1308.284	1588.465	1085.147
	Std	206.0341	448.9127	328.6857	317.7398	260.7149	293.6980	349.2722	292.5697
	Time	0.77	0.79	0.98	0.81	3.40	1.61	0.92	1.16
F21	Mean	883.4526	793.8037	603.0783	887.1336	914.5522	608.4892	638.0682	459.1692
	Std	63.57980	85.17755	64.19765	93.94013	64.08551	60.57776	62.44263	55.04673
	Time	1.07	1.14	1.29	1.12	3.66	1.95	1.24	1.51
F22	Mean	1.39 × 10^4^	9626.454	8046.344	1.09 × 10^4^	1.39 × 10^4^	9342.175	9661.580	6939.285
	Std	623.7961	1480.078	918.6019	1135.879	704.2688	1732.410	1083.247	795.9884
	Time	1.19	1.11	1.40	1.23	3.77	1.96	1.35	1.59
F23	Mean	1611.490	1133.761	820.9687	1413.794	1916.904	971.2375	951.0280	782.8817
	Std	156.8338	96.97311	52.54330	134.8206	122.3561	99.87764	101.2402	60.84830
	Time	1.36	1.31	1.59	1.42	3.84	2.14	1.55	1.81
F24	Mean	1498.221	1128.335	804.2203	1338.020	2125.471	984.8015	1003.564	857.9812
	Std	80.27079	103.4241	43.84820	133.5501	110.1762	90.13087	104.8743	60.08306
	Time	1.47	1.45	1.71	1.54	4.04	2.26	1.66	1.91
F25	Mean	6779.704	7451.468	2327.757	1245.903	7023.516	2449.337	4074.011	682.3776
	Std	1627.463	3851.606	1359.057	192.4039	423.6441	594.1324	2346.966	33.78171
	Time	1.41	1.42	1.62	1.46	3.96	2.34	1.58	1.82
F26	Mean	9547.852	8824.932	5175.204	1.13 × 10^4^	1.11 × 10^4^	5531.918	6907.535	5999.610
	Std	1455.845	1760.854	597.2284	1432.749	543.4698	1480.805	1277.411	1957.419
	Time	1.71	1.61	1.92	1.75	4.30	2.63	1.87	2.10
F27	Mean	1671.716	1165.097	848.4837	1656.802	3582.809	1124.553	1184.41	500.0116
	Std	298.3491	178.4079	96.35885	345.4130	270.3357	208.2852	231.698	0.000195
	Time	1.95	1.95	2.20	2.02	4.60	2.79	2.13	2.36
F28	Mean	5902.595	7109.457	5508.645	2403.685	6397.092	2353.170	5400.214	508.2917
	Std	1279.437	768.2124	593.6870	341.5047	335.9758	653.2835	2199.043	39.60585
	Time	1.75	1.74	1.97	1.81	4.45	2.70	1.92	2.15
F29	Mean	5421.977	4928.239	2062.470	5966.120	9369.429	3161.540	3918.473	1545.224
	Std	1141.993	3064.262	494.6879	1165.999	2320.738	795.2987	1796.600	369.3750
	Time	1.25	1.26	1.47	1.30	3.81	2.20	1.41	1.65
F30	Mean	8.11 × 10^8^	1.33 × 10^9^	3.13 × 10^7^	1.30 × 10^8^	1.42 × 10^9^	7.13 × 10^7^	2.34 × 10^8^	2.49 × 10^5^
	Std	4.13 × 10^8^	1.24 × 10^9^	7.12 × 10^7^	3.44 × 10^7^	5.09 × 10^8^	5.83 × 10^7^	2.73 × 10^8^	6.59 × 10^5^
	Time	2.61	2.47	2.84	2.66	5.32	3.57	2.78	3.00

**Table 8:** The basic information of the engineering optimization problem.

Func.	Name	Expression	Constraint	Variable Scope
F31	Three-bar truss design	$F_{21}\left(x\right)=\left(2\sqrt{2}x_{1}+x_{2}\right)\times l$	$\left(\sqrt{2}x_{1}+x_{2}/\sqrt{2}x_{1}^{2}+2x_{1}x_{2}\right)P-\sigma \leq 0$ $\left(1/\sqrt{2}x_{2}+x_{1}\right)P-\sigma \leq 0$	$0\leq x_{i}\leq 1$ l=100 cm $P=2\mathrm{KN}/{\mathrm{cm}}^{2}$ $\sigma =2\mathrm{KN}/{\mathrm{cm}}^{2}$
F32	Pressure vessel design	$F_{22}\left(x\right)=0.6224x_{1}x_{3}x_{4}+1.7781x_{2}x_{3}+3.1661x_{1}^{2}x_{4}+19.84x_{1}^{2}x_{3}$	$-x_{1}+0.0193x_{3}\leq 0$ $-x_{2}+0.00954x_{3}\leq 0$ $-\pi x_{3}^{2}x_{4}-4\pi x_{3}^{3}/3\leq -1,960,000$ $-x_{4}-240\leq 0$	$0\leq x_{1,\;2}\leq 100$ $10\leq x_{3,\;4}\leq 200$ $x_{1,\;2}$ is multiple of 0.0625.
F33	Tension/compression spring design	$F_{23}\left(x\right)=\left(x_{3}+2\right)x_{2}x_{1}^{2}$	$1-x_{2}^{3}x_{3}/71.785x_{1}^{4}\leq 0$ $4x_{2}^{2}-x_{1}x_{2}/12.566\left(x_{2}x_{1}^{3}-x_{1}^{4}\right)+1/5.108x_{1}^{2}\leq 1$ $1-140.45x_{1}/x_{2}^{2}x_{3}\leq 0$ $x_{1}+x_{2}/1.5-1\leq 0$	$0\leq x_{i}\leq 1$ l=100 cm $P=2\mathrm{KN}/{\mathrm{cm}}^{2}$ $\sigma =2\mathrm{KN}/{\mathrm{cm}}^{2}$

**Table 9:** Statistical results of three engineering optimization problems.

Func.	Algorithm	Optimized Result				Optimization Variable			
		Best	Worst	Std	Mean	*x*_1_	*x*_2_	*x*_3_	*x*_4_
F31	PECSO	263.8959	265.7756	0.4604	264.1986	0.78848	0.40879	-	-
	CSO	264.1046	267.4508	0.7792	265.1001	0.78186	0.42962	-	-
	FCSO	264.3374	267.2195	1.0166	265.2885	-	-	-	-
F32	PECSO	6059.7143	7337.4904	376.0045	6355.1738	0.81250	0.43750	42.09845	176.6366
	CSO	6112.6739	7512.0098	386.8835	6631.4332	0.87500	0.43750	45.19547	141.9197
	FCSO	12272.28	1864.725	2945.724	4803.109	-	-	-	-
F33	PECSO	0.0127	0.0163	0.0008	0.0132	0.05179	0.35902	11.15565	-
	CSO	0.0127	0.0176	0.0011	0.0135	0.05180	0.35935	11.14283	-
	FCSO	0.0128	0.0132	0.0001	0.0130	-	-	-	-

**Table 10:** *D*-*H* parameters of PUMA 560 robot.

NO.	$\mathrm{Link}\;\mathrm{Length}\;(a_{i})$	$\mathrm{Link}\;\mathrm{Torsion}\;\mathrm{Angle}\;(\alpha_{i})$	$\mathrm{Link}\;\mathrm{Offsets}\;(d_{i})$	$\mathrm{Range}\;\mathrm{of}\;\mathrm{Link}\;\mathrm{Joint}\;\mathrm{Angle}\;(\theta_{i})\;$	
				$\theta_{i,min}$	$\theta_{i,max}$
1	0	90°	0	−160°	160°
2	0	0°	0	−245°	45°
3	0.4318 m	−90°	0.1491 m	−45°	225°
4	0.0203 m	90°	0.1331 m	−110°	170°
5	0	−90°	0	−100°	100°
6	0	0°	0	−266°	266°

**Table 11:** Comparison results for inverse kinematics of PUMA 560 robot.

*N*	*T*	PECSO		CSO		BRO	
		Mean	Std	Mean	Std	Mean	Std
100	100	0.00177	0.00241	0.01086	0.00373	1.9146 × 10^−5^	3.7751 × 10^−5^
100	300	7.61 × 10^−7^	1.29 × 10^−6^	0.00880	0.00411	1.0689 × 10^−4^	2.1312 × 10^−5^
200	100	3.46 × 10^−5^	9.43 × 10^−5^	0.00840	0.00351	6.9530 × 10^−5^	2.3740 × 10^−5^
200	300	1.16 × 10^−7^	4.84 × 10^−7^	0.00701	0.00352	6.2040 × 10^−6^	1.5333 × 10^−5^
300	100	1.77 × 10^−7^	3.45 × 10^−7^	0.00778	0.00267	1.8821 × 10^−6^	3.8133 × 10^−6^
300	300	1.32 × 10^−8^	9.58 × 10^−9^	0.00633	0.00326	7.1473 × 10^−7^	2.7865 × 10^−6^
300	500	5.49 × 10^−9^	4.41 × 10^−9^	0.00507	0.00283	1.8914 × 10^−7^	6.4582 × 10^−7^

## Data Availability

The data used to support the findings of this study are included within the article and are also available from the corresponding authors upon request.
